# Introduction to Special Collection on Decolonizing Education in Global Health

**DOI:** 10.5334/aogh.3756

**Published:** 2022-05-24

**Authors:** Carlos A. Faerron Guzmán, Virginia Rowthorn

**Affiliations:** 1 The Graduate School, University of Maryland, Baltimore, 620 W Lexington St, Baltimore, MD 21201, USA

## Abstract

At the heart of the decolonization of global health process lies critical analysis of the interdependent matrices of power dynamics. As characterized by the articles presented in this Special Collection, deep reform of global health can take the form of shifts in leadership structures, priority setting processes, knowledge/cognitive paradigms, power dynamics, financial arrangements, curricular innovation, and policy changes in research, education, and practice.

The curation process of this Special Collection was designed to represent diverse geographies, scales, stakeholders, and themes within the decolonizing global health conversation. The unique perspectives of scholars representing the fields of pharmacy, physiotherapy, medicine, nursing, social work, law, public health, sociology, and bioethics are included in the collection. The premise of the Special Collection is the understanding that meaningful progress toward decolonization must come from within the institutions that built the field of global health in the first place, and doing so will require deep reflection on the role different disciplines – working both alone and collaboratively – can and should play to advance decolonization.

On May 25th and 26th, 2021, the University of Maryland Baltimore hosted a two-day virtual summit to discuss the importance of decolonizing global health education. Understanding that the topic of decolonization cannot be tackled superficially or without the critical input of stakeholders who have borne the brunt of the impacts of colonization, the summit was designed to capture the voices of students and trainees from around the world across multiple professions. To facilitate the conversation, a curated pre-summit mini-curriculum was used. This curriculum included a reading list, multimedia resources, reflective exercises for students, and two pre-summit virtual workshops with students and faculty from the UMB community and global partners.

During the summit, speakers and panelists from around the world with varying backgrounds, experiences, and perspectives came together to break down their definitions of decolonization and the innumerable barriers in existence that keep essential change from occurring. Summit participants mainly explored how racism, white supremacy, and other forms of coloniality impact their professional practice (a summary of these explorations from the summit is represented in ***Figure 1***). The grey boxes represent the stakeholders involved in this process, the blue boxes represent the means by which coloniality is enacted, and the arrows represent the directionality of the colonial process.

**Figure 1:**
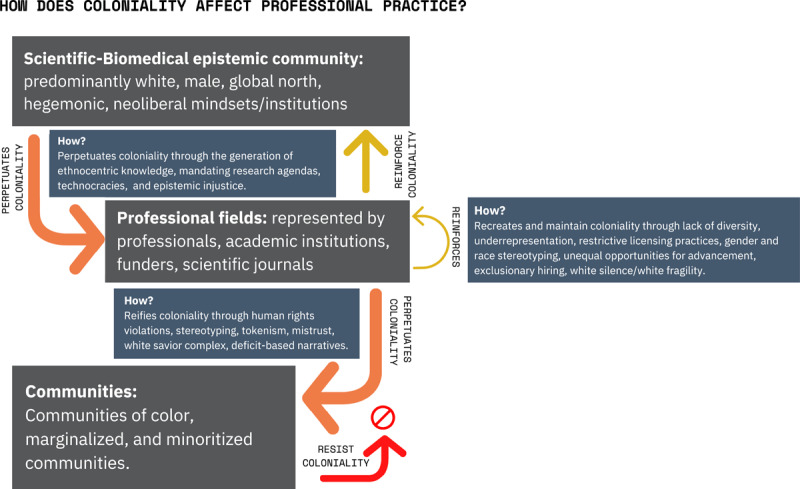
Coloniality, professional practice, and its impact on communities.

The summit served as an opportunity for participants to reflect on and solidify the next steps to work towards a future decolonized, equitable, diversified, and culturally competent system of higher education. The articles in this two-part Special Collection of the *Annals of Global Health* were developed by the international groups of students and faculty who participated in the summit. This summit was embedded as part of the growing movement and effort to decolonize global health [1,2,3].

The process of global health decolonization is still not well defined, and whether global health as a field can survive said process is still an ongoing debate [4]. What is clear to many is that the current field of global health, represented by institutions and practitioners, still lacks the necessary tools, understanding, and will to deconstruct and reshape its own structure critically [5].

Despite advances in multiple aspects of the decolonization agenda, the entrenched nature of the power asymmetries within global health is still represented by ongoing imbalances in whose voices count, what systems of knowledge are valued, who gets to set the priorities, and who holds the financial resources. Perceived superiority, moral and intellectual, topped with the geopoliticization (i.e., the use of global health as a foreign policy instrument), continuously reify the vertical and exclusionary practice of global health. Without critical introspection and acknowledgment of these dynamics, global health will continue to represent a legacy of its colonial past and justification.

At the heart of the decolonization of global health processes lies critical analysis of the interdependent matrices of power dynamics (i.e., intersectional analysis) [5], a recurrent theme within this Special Collection. As characterized by the articles presented here, deep reform of global health can take the form of shifts in leadership structures, priority setting processes, knowledge/cognitive paradigms, power dynamics, financial arrangements, curricular innovation, and policies changes in research, education, and practice.

We developed this Special Collection around the theme of decolonization broadly defined, emphasizing how coloniality and neo-colonial thought are woven through and still affect higher education. Through this collection, we have provided an opportunity for summit participants (faculty, researchers, practitioners, students, and administrators) to express their vision of what a decolonized global health field looks like. Our curation process of this Special Collection was designed to represent diverse geographies, scales, stakeholders, and themes within the decolonizing global health conversation. The unique perspectives of scholars representing the fields of pharmacy, physiotherapy, medicine, nursing, social work, law, public health, sociology, and bioethics are included in the collection. The premise of the Special Collection is the understanding that meaningful progress toward decolonization must come from within the institutions that built the field of global health in the first place and doing so will require deep reflection on the role different disciplines – working both alone and collaboratively – can and should play to advance decolonization.

The motivation for this special issue comes at a time when prevailing orthodoxies are being challenged in global health, as evidenced by the rise in the literature addressing global health and decolonization (***Figure 2***). However, many more spaces for discussion are still needed, especially those led by voices of the “majority world.”

**Figure 2:**
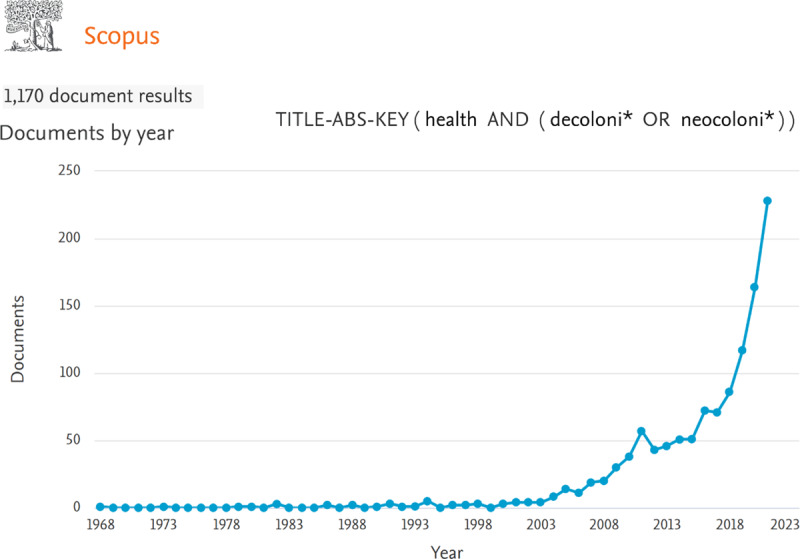
**Publications per year on Decolonization and Health.** The Scopus database was used to retrieve documents in peer-reviewed journals. Search strategy TITLE-ABS-KEY (health) AND TITLE-ABS-KEY(decoloni* OR neocoloni*).

Although each article is written from a distinct vantage point, with a different focus, and borrows from different experiences and voices, the editorial team discerned distinct themes threaded through the two volumes of this Special Collection. The first explores queries and approaches that address the structural issues in decolonizing global health (i.e., high-level perspectives). The second brings perspectives and case studies of global health practitioners and their approaches in decolonizing global health (i.e., decolonization in practice).

We hope that the contributions in the two components of this Special Collection will ground through case studies, reflections, and concrete recommendations, the current conceptual debate around “what does decolonizing global health look like?” Although this special issue is not a comprehensive coverage of the challenges and disciplines that need to be included in the broad task of decolonizing global health (neither was that our intent), we hope the contributions in this special issue jointly advance the decolonization movement to reach our common goals.

Furthermore, we expect that the articles in this special edition help the reader navigate ongoing questions in the decolonizing global health movement, such as: What are the concrete ways that university leadership can move forward with decolonizing global health? How can global health educators reshape their curricula? How can different stakeholders create more equitable international partnerships that represent the values of liberal education? What role can learners play in the process of decolonizing global health and global health education? How can research agendas equitably represent the needs of stakeholders that historically have had less participation? What role do professional codes of ethics and legal and policy frameworks play in the decolonizing process?

To our knowledge, this is the first effort to bring together diverse perspectives on the process of decolonizing global health, primarily focused on education. Neither prescriptive nor definitive, the contributions here fill only a small portion of the existing gaps towards a decolonized global health field. We hope that more interdisciplinary research is motivated by this work and by future publication in this vein. Finally, we would like to thank the *Annals of Global Health* editorial team and the reviewers for their constructive criticisms. This Special Collection is undoubtedly more robust thanks to their contributions.
